# Identification of Orphan Genes in Unbalanced Datasets Based on Ensemble Learning

**DOI:** 10.3389/fgene.2020.00820

**Published:** 2020-10-02

**Authors:** Qijuan Gao, Xiu Jin, Enhua Xia, Xiangwei Wu, Lichuan Gu, Hanwei Yan, Yingchun Xia, Shaowen Li

**Affiliations:** ^1^Anhui Province Key Laboratory of Smart Agricultural Technology and Equipment, Anhui Agriculture University, Hefei, China; ^2^State Key Laboratory of Tea Plant Biology and Utilization, Anhui Agricultural University, Hefei, China; ^3^School of Resources and Environment, Anhui Agricultural University, Hefei, China; ^4^School of Information and Computer Science, Anhui Agricultural University, Hefei, China; ^5^Key Laboratory of Crop Biology of Anhui Province, Anhui Agricultural University, Hefei, China

**Keywords:** unbalanced dataset, ensemble learning, orphan genes, XGBoost model, two-class

## Abstract

Orphan genes are associated with regulatory patterns, but experimental methods for identifying orphan genes are both time-consuming and expensive. Designing an accurate and robust classification model to detect orphan and non-orphan genes in unbalanced distribution datasets poses a particularly huge challenge. Synthetic minority over-sampling algorithms (SMOTE) are selected in a preliminary step to deal with unbalanced gene datasets. To identify orphan genes in balanced and unbalanced *Arabidopsis thaliana* gene datasets, SMOTE algorithms were then combined with traditional and advanced ensemble classified algorithms respectively, using Support Vector Machine, Random Forest (RF), AdaBoost (adaptive boosting), GBDT (gradient boosting decision tree), and XGBoost (extreme gradient boosting). After comparing the performance of these ensemble models, SMOTE algorithms with XGBoost achieved an F1 score of 0.94 with the balanced *A. thaliana* gene datasets, but a lower score with the unbalanced datasets. The proposed ensemble method combines different balanced data algorithms including Borderline SMOTE (BSMOTE), Adaptive Synthetic Sampling (ADSYN), SMOTE-Tomek, and SMOTE-ENN with the XGBoost model separately. The performances of the SMOTE-ENN-XGBoost model, which combined over-sampling and under-sampling algorithms with XGBoost, achieved higher predictive accuracy than the other balanced algorithms with XGBoost models. Thus, SMOTE-ENN-XGBoost provides a theoretical basis for developing evaluation criteria for identifying orphan genes in unbalanced and biological datasets.

## Introduction

The process of identifying orphan genes is an emerging field. Orphan genes play critical roles in the evolution of species and the adaptability of the environment (Davies and Davies, 2010; Donoghue et al., 2011; Huang, 2013; Cooper, 2014; Gao et al., 2014). In most plant species, orphan genes make up about 10–20% of the number of genes (Khalturin et al., 2009; Tautz and Domazet-Loso, 2011), and each species has a specific proportion of orphan genes (Khalturin et al., 2009; Arendsee et al., 2014), Many attempts have been made to identify orphan genes in multiple species or taxa and to analyze their functions. The whole genome and transcriptome sequences of many species have been published, including those of *Arabidopsis thaliana* (Arabidopsis Genome Initiative, 2002), O*ryza sativa* (Goff et al., 2002), Populus (Tuskan et al., 2006), and the discovery of orphan genes among these sequences has helped to clarify the special biological characteristics and environmental adaptability of angiosperm. For example, the *A. thaliana* orphan genes qua-quine starch (*QQS*) alter the carbon and nitrogen content of the plant, increasing the protein content and decreasing the starch content (Li et al., 2009; Arendsee et al., 2014); the wheat, *TaFROG* (*Triticum aestivum* fusarium resistance orphan gene) contributes to disease resistance genes for crop-breeding programs (Perochon et al., 2015); and the rice orphan gene *GN2* (GRAINS NO. 2) can affect plant height and rice yield (Chen et al., 2017).

Currently, orphan genes are detected mainly by comparison of genome and transcriptome sequences of related species using BLAST (Blast-Basic Local Alignment Search Tool; Altschul et al., 1990; Tollriera et al., 2009). However, this approach requires large server resources and time, and common problems with complexity and timeliness occur (Ye et al., 2012).

Computational technology and machine learning (ML) algorithms are widely used in the detection of orphan genes in big datasets. The method of ML can be used to make two kinds of field classification from an enormous genome dataset (Libbrecht and Noble, 2015; Syahrani, 2019). Orphan genes are widely distributed in plant species and generally exhibit significant differences in gene length, the number of exons, GC content, and expression level compared to protein-coding genes (Donoghue et al., 2011; Neme and Tautz, 2013; Yang et al., 2013; Arendsee et al., 2014; Xu et al., 2015; Ma et al., 2020). In systems biology, traditional classification methods, such as Support Vector Machines (SVMs; Zhu et al., 2009) or Random Forest (RF; Pang et al., 2006; Dimitrakopoulos et al., 2016) have been applied in the classification scheme. More recently, ensemble classification algorithms have achieved remarkable results in the fields of biology and medicine (Chen and Guestrin, 2016).

Additionally, the number of orphan genes is much less than the numbers of non-orphan gene datasets, therefore unbalanced datasets pose significant problems for developers of classifiers. The original method of over-sampling and under-sampling (Drummond and Holte, 2003; Chen and Guestrin, 2016) can help address the problems of an unbalanced dataset (Weiss, 2004; Zhou and Liu, 2006). In over-sampling methods, the synthetic minority over-sampling technique (SMOTE) (Demidova and Klyueva, 2017) can add new minority class examples, but the deleted information of majority samples may contain representative information of the majority class. Then, the improved SMOTE which combines with edited nearest neighbors (SMOTE-ENN) algorithm (Zhang et al., 2019), is used in the K-nearest neighbor (KNN) method to classify the sampled dataset, by the theory of over-sampling and under-sampling.

The bagging and boosting methods are two important approaches to ensemble learning (Breiman, 1996) that can improve the accuracy of a model significantly. The boosting family algorithm adaptively fits a series of weak models and combines them. Because the number of minority samples in an unbalanced dataset is small, they are easily misclassified, so the results of the previous classifier determine the parameters of the later model and let the next classifier focus on training the last misclassified sample. Therefore, the Boosting family algorithm pays more attention to samples that are difficult to classify, which can effectively improve the prediction accuracy.

In the study described in this manuscript, over-sampling and under-sampling algorithms were introduced to clean up unbalanced data (Chawla et al., 2002). Representative serial classified algorithms of the Boosting family are AdaBoost (adaptive boosting), GBDT (gradient boosting decision tree), XGBoost (extreme gradient boosting), and the representative parallel classified algorithm are SVM and RF. The performance of these five classification models with over-sampling SMOTE is better than those with single classifiers. The relevant features of the whole gene sequencing of *A. thaliana* were designed as a model for the identification and prediction of orphan genes. The result could show that balancing algorithms play a more effective guiding role in identifying the orphan genes in a species.

## Materials and Methods

### Data Processing Method for Unbalanced Data

Data preprocessing is the first step for data mining and affects the result. Preprocessing includes data discretization, missing values, attribute coding, and data standard regularization. In practice, each industry has unique data characteristics, so different methods are used to analyze the data and perform preprocessing.

The processing of unbalanced data describes classes with obviously uneven distribution. The traditional method used random over-sampling to increase the number of small-class samples to achieve a consistent number. Because this method achieves balance by a single random over-sampling strategy of copying data, the added repeated data will increase the complexity of data training and induce over-fitting.

To deal with the problem of unbalanced data classification, some algorithms have been used effectively to improve the performance of classification. Common methods for processing datasets included mainly: over-sampling and under-sampling, or a combination of under-sampling and over-sampling.

### Over-Sampling SMOTE and Borderline SMOTE

To solve the problem of over-fitting associated with unbalanced data when the learning information is not generalized, Chawla et al. (2002) proposed the SMOTE algorithm for preprocessing over-sampling data of synthetic minority categories. SMOTE was designed based on a random over-sampling method in the feature space. By analyzing data with few categories, many new data are generated by linear interpolation and added to the original data set. SMOTE first selects each sample from the minority samples successively as the root sample for the synthesis of the new sample. Then according to the up-sampling rate n, SMOTE randomly selects one of *K* (*K* is generally odd, such as *K* = 5) neighboring samples of the same category, which is used as an auxiliary sample to synthesize a new sample and repeated *n* times. Finally, linear interpolation is performed between the sample and each auxiliary sample to generate n synthesized samples. The basic flow of the algorithm is:

(i) Find *K* samples of the nearest neighbor for each sample x_i_, whose label is “1”;
(ii) A sample x_j_ belonging with few categories is selected randomly from *K*;
(iii) Linearly interpolate randomly between x_i_ and x_j_ to construct a new minority sample.

The SMOTE algorithm effectively solves the problem of over-fitting caused by the blind replication of random over-sampling techniques. However, the selection of the nearest neighbor sample in step 1 exits is purposeless. Users need to determine the number of *K* values of the neighbor samples themselves, so it is difficult to determine the optimal value. Additionally, the newly synthesized samples may fall into the sample area labeled “0,” which confuses the boundaries between them and interferes with the correct classification of the data.

Therefore, to address these two problems, Wang et al. (2015) proposed Borderline SMOTE (an over-sampling method in unbalanced datasets learning), which is an improved over-sampling algorithm based on SMOTE. By finding suitable areas that can better reflect the characteristics of the data to be interpolated, the problem of sample overlap can be solved. The Borderline SMOTE algorithm uses only a few samples on the boundary to synthesize new samples, thereby improving the internal distribution of samples.

### Adaptive Synthetic Sampling

Adaptive Synthetic Sampling adaptively generates different numbers of sampling samples according to data distribution (He et al., 2008). The basic flow of the algorithm is below:

(i) Calculate the number of samples to be synthesized, as follows:G = (*m*_l_−*m*_s_)×β, where *m*_l_ is the number of majority samples, and*m*_s_ is the number of minority samples. If β = 1, the number of positive and negative samples is the same after sampling, indicating that the data is balanced at this time.
(ii) Calculate the number of K nearest neighbor value of each minority sample, Δ is the number of majority samples in the K neighbors, the formula is as follows: *r*_i_ = Δ_i_/*K*, where Δ_i_ is the number of majority samples in K nearest neighbors, *i = 1,2,3…….*,*m*_s_
(iii) To normalize *r*_i_, the formula is $\hat{r}=r_{\text{i}}/\sum_{i=1}^{m_{\text{s}}}r_{\text{i}}$
(iv) According to the sample weights, calculate the number of new samples that need to be generated for every minority sample. The formula is $\text{g}=\hat{r}\times G$.

Select one sample from the K neighbors around each data with the label “1” to be synthesized, calculate the number to be generated according to g the formula*s*_i_ = *x*_i_ + (*x*_zi_−*x*_i_)×λ, where *s*_i_ is the synthetic sample, *x*_i_ is the *i*th minority samples, and *x*_zi_ is a random number of the minority sample λ∈ [0,1] selected from the K nearest neighbors of *x*_i_.

### Combining Algorithms

**FIGURE 1:**
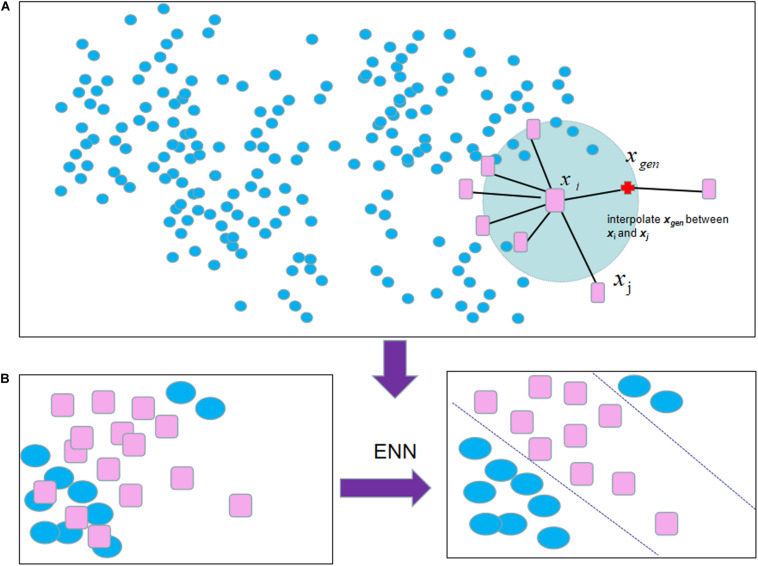
The process of SMOTE-ENN algorithm: **(A)** SMOTE selected each sample from the minority samples successively as the root sample for the synthesis of the new sample. **(B)** The following result was obtained by employing ENN to eliminate noise samples when the process of SMOTE is caused.

Apart from using a single under-sampling or over-sampling method, two resampling methods can be combined. For example, SMOTE-ENN (Zhang et al., 2019), ENN is an under-sampling method focusing on eliminating noise samples, which is added to the pipeline after SMOTE to obtain cleaner combined samples. For each combined sample, its nearest-neighbors are computed according to the Euclidean distance. These samples will be removed whose most KNN samples are different from other classes (shown in Figure 1).

SMOTE-Tomek (Batista et al., 2004) also combine SMOTE with Tome-links (Tomek), a data cleaning method to handle the overlapping parts, which are difficult to classify for a few classes and most surrounding samples. A Tome link can be defined as follows: given that sample x and y belong to two classes, and be the distance between *x* to *y* as *d* (*x*,*y*). If there is not a sample z, such as *d* (*x*,*z*) < *d* (*x*,*y*) or *d* (*y*,*z*) < *d* (*x*,*y*), *A* (*x*,*y*) pair is called a Tome link.

### Ensemble Learning Methods

The main idea of the ensemble learning algorithm is to construct multiple classifiers with weak performance and use a certain strategy to combine them into a classifier with strong generalization performance. Consequently, the performance of the ensemble is better than that of a single classifier.

This study created two classification models for unbalanced datasets and used Python to build five integrated learning models of SVM, RF, AdaBoost, GBDT, and XGBoost and conducted comparative experiments to find the optimal model. XGBoost performed best in the classification, Five kinds of balanced data learning methods of resampling: SMOTE, BSMOTE, ADASYN, SMOTE-ENN, and SMOTE-Tomek, were then combined with XGBoost to build an ensemble model that produced excellent classification results (Lemaitre et al., 2017; Wu et al., 2018).

XGBoost was modified by adding regular items to the GBDT algorithm that can predict the orphan gene binary classification problem and increase the calculation speed. XGBoost uses the gradient boosting algorithm of the based learner classification and regression tree (CART) to calculate the complexity of the leaf nodes of each tree and uses the gradient descent algorithm to minimize the loss for finding the optimal prediction score, thus avoiding over-fitting the learned model and effectively controlling the complexity of the model (Chen and Guestrin, 2016).

The derivation process is as follows:

(i) Objective function: $\text{obj}\,\left(\theta \right)=\sum_{\text{i}}^{n}l\,\left(y_{\text{i}},\hat{y_{\text{i}}}\right)+\sum_{k=1}^{K}\Omega \,\left(f_{k}\right)$
(ii) Using the first and second derivatives, the Taylor formula expands:

$${\text{obj}}^{\left(t\right)}=\left[\sum_{\text{i}}^{n}l\,\left(y_{\text{i}},{\hat{y_{\text{i}}}}^{t-1}\right)+g_{\text{i}}\,f_{t}\,\left(x_{\text{i}}\right)\right]+\Omega \,\left(f_{t}\right)+c\,o\,n\,s\,t\,a\,n\,t$$

(iii) Measuring the complexity of the decision tree as:$\Omega \,\left(\text{f}\right)=\gamma \,T+\frac{1}{2}\,\lambda \,\sum_{j=1}^{T}w_{\text{j}}^{2}$, where T is the number of leaf nodes in the decision tree, and w is the prediction result corresponding to the leaf node.
(iv) Substituting the above two steps into the objective function (1), it is organized as:

$${\text{obj}}^{\left(t\right)}\approx \sum_{i=1}^{n}\left[g_{\text{i}}\,w_{q(x_{i)}}+\frac{1}{2}\,\left(h_{\text{i}}\,w_{q\,\left(x_{\text{i}}\right)}^{2}\right)\right]+\gamma \,T+\frac{1}{2}\,\sum_{j=1}^{T}w_{\text{j}}^{2}=\sum_{j=1}^{T}\left[G_{\text{j}}\,w_{\text{j}}+\frac{1}{2}\,\left(H_{\text{j}}+\lambda \right)\,w_{\text{j}}^{2}\right]+\gamma \,\text{T}$$

(v) Then, *I*_j_ = {*i*|*q*(*x*_i_) = *j*}, represents the sample set belonging to the j-th leaf node.

$$G_{\text{j}}=\sum_{i\in I_{\text{j}}}g_{\text{i}},H_{\text{j}}=\sum_{i\in I_{\text{j}}}h_{\text{i}},$$

(vi) To minimize the objective function, let the derivative be 0 and find the optimal prediction score for each leaf node:

$$w_{j}^{{}^{*}}=-\frac{G_{j}}{H_{j}+\lambda }$$

(vii) Substitute the objective function again to get its minimum value:

$${\text{obj}}^{\left(t\right)}=-\frac{1}{2}\,\sum_{J=1}^{T}\frac{G_{j}^{2}}{H_{j}+\lambda }+\gamma \,T$$

(viii) Find the optimization goal of each layer of the build tree through obj to find the optimal tree structure, and split the left and right subtrees as:

$$\text{Gain}\left(\phi \right)=\frac{1}{2}[\frac{{\left(\sum_{i\subseteq I_{\text{L}}}g_{\text{i}}\right)}^{2}}{\sum_{i\subseteq I_{\text{L}}}h_{\text{i}}+\lambda }+\frac{{\left(\sum_{i\subseteq I_{R}}g_{\text{i}}\right)}^{2}}{\sum_{i\subseteq I_{R}}h_{\text{i}}+\lambda }-\frac{{\left(\sum_{i\subseteq I}g_{\text{i}}\right)}^{2}}{\sum_{i\subseteq I}h_{\text{i}}+\lambda }]-\gamma$$

### Confusion Matrix

The confusion matrix (error matrix) is a matrix table (shown in Table 1) that is used to judge whether a sample is 0 or 1 and reflects the accuracy of classification. The results of the classification model are analyzed using four basic indicators: true positive (TP), true negative (TN), false positive (FP), and false negative (FN). The prediction classification model that gives the best results will have a large number of TPs and TNs and a small number of TPs and TNs.

**TABLE 1:** Binary confusion matrix.

	Real positive	Real negative
Predict positive	TP	FP
Predict negative	FN	TN

(i) True positive (TP): the actual value of the model is the orphan genes, so the model predicts the number of orphan genes.
(ii) False positive (FP): the actual value of the model is the orphan gene, but the model predicts the number of non-orphan genes.
(iii) False negative (FN): the true value of the model is non-orphan genes, so the model predicts the number of orphan genes.
(iv) True negative (TN): the true value of the model is non-orphan genes, but the model predicts the number of non-orphan genes.

### Recall, Precision, and F1 Value as Performance Indicators

A large number of confusion matrix statistics make it difficult to measure the pros and cons of a model. Therefore, we added using Recall, Precision, and F1-score, as performance indicators to better evaluate the performance of the model:

(i) Recall rate (accuracy rate of positive samples):

$$\text{Recall}=\frac{\text{TP}}{\text{TP}+\text{FN}}$$

(ii) Precision (precision rate of positive samples):

$$\text{Precision}=\frac{\text{TP}}{\text{TP}+\text{FP}}$$

(iii) F1-score value:

$${\mathrm{F1}}_{\text{SCORE}}=\frac{2\,\text{PR}}{\text{P}+\text{R}}$$

### ROC Curve and AUC Value

The receiver operating characteristic (ROC) curve reflects the probability of identifying correct and wrong results according to different thresholds. The curve passes (0, 0) and (1, 1), and the validity of the model is generally determined by the diagonal of the curve in the upper left section of the graph.

The AUC value is the value of the area under the ROC curve, which is generally between 0.5 and 1. The quantized index value can better compare the performance of the classifiers: a high performance classifier AUC value is close to 1.

## Results

### Collating Feature Data of Orphan and Non-orphan Genes

The whole genome data of the angiosperm *A. thaliana* were obtained from The Arabidopsis Information Resource (TAIR8) dataset ftp://ftp.arabidopsis.org/home/tair/Genes/TAIR8_genome_release, which contained a total of 32825 gene sequences. The known orphan genes of *A. thaliana* downloaded from the public website https://www.biomedcentral.com/content/supplementary/1471-2148-10-%2041-S2.TXT (Lin et al., 2010). The protein sequences and coding sequences were downloaded from TAIR. GC percent, protein length, molecular mass, protein isoelectric point (pI), average exon number were selected.

The six features of the protein and coding sequences were recorded as V1-V6 (Perochon et al., 2015; Shah, 2018; Ji et al., 2019). The class of orphan genes is recorded as a *Class* problem, where the label of orphan genes is recorded as 1 and the non-orphan genes are recorded as 0, combined with V1–V6 features (Ji et al., 2019; Li et al., 2019).

### Analyzing Orphan and Non-orphan Gene Dataset

There were 32825 samples in the gene datasets, but only about 4.08% of them were orphan genes, so the distribution of orphan and non-orphan samples was uneven. We evaluated whether the models can identify the orphan genes. For traditional ML classification algorithms, the premise is that the amount of data between categories is balanced, or that the cost of misclassification for each category is the same. Therefore, the direct application of many algorithms leads to more predictions being made for the category with a larger number.

To solve the problem, of unbalanced data sets, we first used over-sampling to copy small sample data, which increased the number of categories with fewer samples. This method balanced the numbers of orphan and non-orphan samples to improve the learning ability of the classifier. The random sampling method was used to divide the samples into training and testing sets with a ratio of 8:2 which is the same ratio as the original dataset (Table 2).

**TABLE 2:** Training and testing datasets used to design and evaluate the model classifiers.

Class	Train dataset	Test dataset	Original dataset
None-orphan genes	24833	6208	31041
Orphan genes	1427	357	1784

The training set was used to design the model, and the test set was used to test the performance of the model. The Precision, Recall, F1, and AUC evaluation indicators were used to compare the model classifiers to determine the effectiveness of the models and select the best model.

**FIGURE 2:**
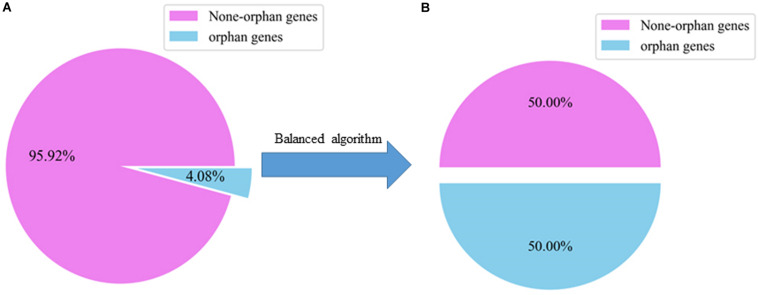
Ratio of orphan to non-orphan and orphan genes. **(A)** The distribution of an unbalanced dataset in the original *A. thaliana.* **(B)** The distribution of *A. thaliana* datasets are balanced after using a balanced algorithm.

We used SMOTE to balance the numbers of orphan and non-orphan genes in the original *A. thaliana* gene dataset shown in Figure 2.

### Training Model Using Ensemble Learning Methods

Among the ensemble learning methods, some members of the Boosting family, such as AdaBoost., GBDT, XGBoost, can be used to train classifying models, which can save the compute time remarkably (Table 3).

**TABLE 3:** Compute time compared among Adaboost, GBDT, XGBoost models with SMOTE algorithm.

Traing model	Time (s)
AdaBoost	11.7
GBDT	10.3
XGBoost	0.3

Two parameters, train_node and learning_rate were considered to reduce the complexity in modeling. However, selecting the best parameters for the ensemble learning algorithms is important to avoid an over-fitting problem. For this study, we set the learning_rate as 0.01, 0.1, and 0.2 and train_node as 100, 150, 200 to compute the F1 score.

AdaBoost, GBDT, XGBoost with the two parameters are used to classify the samples in the training and testing datasets (Table 2). The results are shown in Table 4.

**TABLE 4:** F1 scores of GBDT, Adaboost, XGBoost models with the SMOTE algorithm on test datasets.

n_estimator	Learning_rate	Testing Algorithm (%)		
		GBDT	AdaBoost	XGBoost
200	0.2	90	87.6	93
200	0.1	89	88	92
200	0.01	87	87.4	88
150	0.2	90	87.9	93
150	0.1	89	87.4	91
150	0.01	87	87.4	88
100	0.2	89	87.5	92
100	0.1	88	87.5	90
100	0.01	87	87.5	88

Overall, the XGBoost with SMOTE performed better than AdaBoost and GBDT models with SMOTE.

### Performance of Different Models With Balanced and Unbalanced Datasets

Five models, SVM, RF, GBDT, AdaBoost, and XGBoost were used as baseline classifiers to distinguish orphan and non-orphan genes in the unbalanced and balanced *A. thaliana* gene datasets. The results are shown in Table 5.

**TABLE 5:** Performance of models in distinguishing orphan vs. non-orphan genes in *A. thaliana* gene balanced and unbalanced datasets with 8:2 training-testing ratios.

Best Model	Unbalanced datasets (%)					Balanced datasets (SMOTE) (%)				
	Accuracy	Precision	Recall	F1	AUC	Accuracy	Precision	Recall	F1	AUC
SVM	97	78	47	58	74	83	83	83	83	88
RF	96	47	58	52	93	84	77	98	86	95
GBDT	96	60	59	60	94	87	87	87	87	94
Adaboost	97	56	73	45	93	87	87	86	89	95
XGBoost	97	81	50	62	94	92	91	95	93	97

Overall, the five models produced better results with the balanced datasets. However, the accuracy of the models with the balanced datasets was lower than with the unbalanced dataset, which indicates the classification of orphan genes was towards the majority samples of non-orphan genes. These results clearly show that designing models using unbalanced datasets will lead to significant inaccuracies, which cannot identify orphan genes VS non-orphan genes precisely. This indicates the importance of using a balancing algorithm to balance datasets in the first step of the classification process.

**FIGURE 3:**
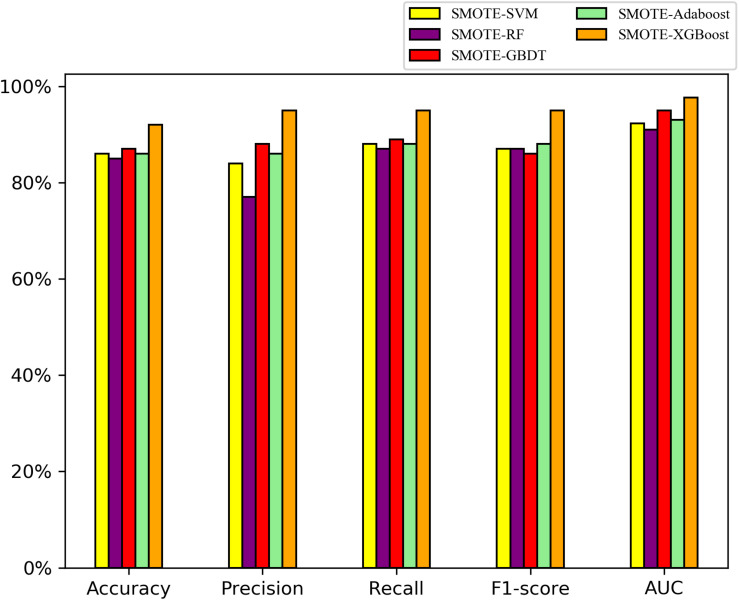
Performance indices of five classifiers model with the SMOTE algorithm on the testing dataset to distinguishing orphan and non-orphan genes after balancing the distribution of *A. thaliana* gene dataset.

On the balanced *A. thaliana* gene dataset, the performance indices of five classifier models on the testing datasets are shown in Figure 3. Overall, the ensemble models were better than the single classifiers, as determined by the performance indicators, among them, the AUC and precision values of XGBoost, GBDT, AdaBoost with SMOTE were higher than SVM, RF with SMOTE algorithm. Particularly, XGBoost with SMOTE produced the highest results among all classifier models (*t*-test, *P* < 0.05). In particular, the F1 value indicates that the XGBoost model can distinguish orphan genes and non-orphan genes precisely.

**FIGURE 4:**
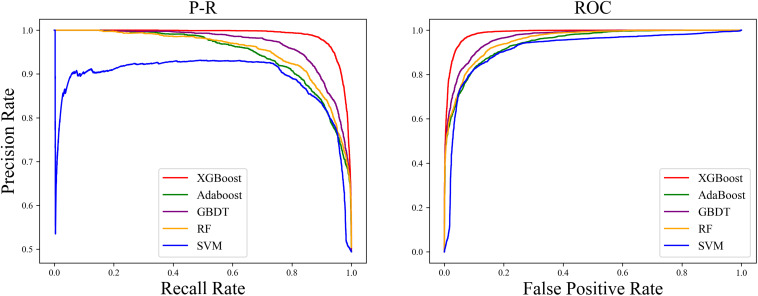
Precision-Recall (PR) curve and ROC curve “True” area and for the five classifiers with an unbalanced dataset.

We found that the ROC curve of SMOTE-XGBoost completely wrapped the ROC curves of the other methods, and the Precision-Recall (PR) curve confirmed that XGBoost produced the best performance among the five balancing algorithm methods (Figure 4).

The PR curve (Figure 4) indicated that when the classification threshold was near 1, all the samples were classified as non-orphan genes, and the Precision and Recall values were 0 at this time. When the classification threshold was 0.9, there were no FPs, so the Precision was 1, which means all the genes were classified as orphans. Because the number of TPs was small, the Recall was small and the Precision value declined continually. When the threshold declined to 0, all the samples were classified as non-orphan genes, meaning that the Precision will not be 0, because there were no FNs, and the Recall value was 1. This indicates that the prediction result is reasonable.

### Performance of XGboost With Different Balanced Algorithm Methods

**FIGURE 5:**
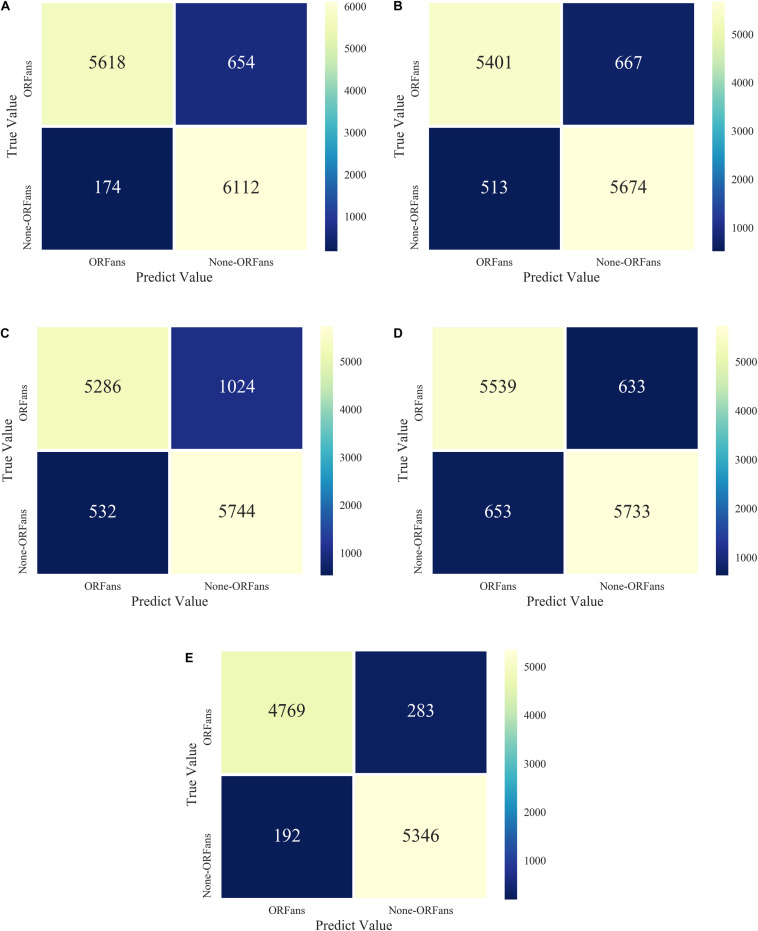
Model confusion matrices for XGBoost combined with five over-sampling models: **(A)** ADASYN-XGB; **(B)** SMOTE-Tomek-XGB; **(C)** BSMOTE-XGB; **(D)** SMOTE-XGB; **(E)** SMOTE-ENN-XGB.

We also tested five different models, XGBoost combined with a balanced algorithm including SMOTE, BSMOTE, ADASYN, SMOTE-Tomek, SMOTE-ENN, to further explore the result of the unbalanced datasets. The results of the confusion matrices of five models are shown in Figure 5. The performance of the SMOTE-ENN-XGBoost model is better and the predicted value is higher, which indicates fewer incorrect classifiers.

The performance indices of the five balanced algorithms with ensemble XGBoost classifiers models are shown in Table 6. The ensemble SMOTE-ENN-XGB model had the highest among the other ensemble models to predict orphan genes (ORFans).

**TABLE 6:** Performance indices of the ensemble of composite XGBoost classifiers.

Evaluation value	ADASYN-XGB (%)	BSMOTE-XGB (%)	SMOTE-XGB (%)	SMOTE-ENN-XGB (%)	SMOTE-Tomek-XGB (%)
Accuracy	85	92	88	95	89
Precision	83	89	87	94	88
Recall	89	97	89	95	90
F1	86	93	88	95	89
AUC	92	97	95	98	96

Therefore, the SMOTE-ENN-XGBoost model is used to classify and analyze the orphan genes in unbalanced datasets and applied to the actual predictions.

## Discussion

Our research indicates that in the classification of orphan vs Non-orphan genes the ML method is preferred because the traditional biological method is time-consuming and labor-intense. Since the orphan genes of plant species have similar characteristics, we selected 6 features of the *A. thaliana* dataset to build training and testing models (Donoghue et al., 2011).

The datasets of orphan genes and non-orphan genes are often unbalanced, which tends to produce a bias towards majority samples. To overcome this problem, we combined over-sampling and under-sampling algorithms, making the trained model with balanced datasets, which improves the generalization ability of the model, and eventually, the precision, recall, F1, and AUC for the test set are significantly increased. To further compare the result of the evaluation, the balanced algorithm combines classifying learning algorithms, RF, SVM, Adaboost, GBDT, XGBoost, which have similar improved results. Furthermore, the boosting methods containing Adaboost, GBDT, XGBoost have a better performance than those that use RF and SVM. Thus, ensemble boosting learning models are an important method in advancing the identification of orphan genes and non-orphan genes in unbalanced datasets. At the same time, the same training node and learning_rate parameters were automatically used for parallel computing among the boosting methods, which revealed that the XGBoost model was more practical than other models for classifying orphan genes. In particular, since it saves time and labor, classifying orphan versus non-orphan genes experimentally in this way could benefit this field and future studies.

To increase the precision of these ensemble models, we compared five different balanced algorithms including SMOTE, BSMOTE, ADASYN, SMOTE-Tomek, SOMTE-ENN combing with XGBoost models. SMOTE-ENN with XGBoost has a better evaluation result, especially the value of Recall. In this paper, we propose the SMOTE-ENN-XGBoost model for efficiently identifying unbalanced datasets of orphan genes. We built the SMOTE-ENN-XGBoost model to classify genes by predicting 0 or 1 values. The results showed that the ensemble classifiers method classified the orphan and non-orphan genes more precisely than the single classifiers, and among the five ensemble models with XGBoost, the SMOTE-ENN-XGBoost model performed best.

This study provides a new method for the identification of unbalanced datasets of orphan genes, which can be applied in the classification of unbalanced biological datasets. Meanwhile, the method can support the evolution of species.

## Data Availability Statement

The datasets presented in this study can be found in online repositories. The names of the repository/repositories and accession number(s) can be found in the article/Supplementary Material.

## Author Contributions

QG and XJ: development of methodology. HY and YX: sample collection. QG, XJ, EX, and XW: analysis and interpretation of data. QG, XJ, LG, and SL: writing, review, and revision of the manuscript. All authors contributed to the article and approved the submitted version.

## Conflict of Interest

The authors declare that the research was conducted in the absence of any commercial or financial relationships that could be construed as a potential conflict of interest.
